# Case Report: Immunotherapy-induced myocarditis requiring pacemaker insertion in an older adult. what happens if we rechallenge?

**DOI:** 10.3389/fonc.2026.1565918

**Published:** 2026-02-03

**Authors:** Wassim Assaad, Nour El Meski, Omar Fakhreddine, Tarek El Halabi, Marwan Refaat, Firas Kreidieh

**Affiliations:** 1 Department of Internal Medicine, Division of Cardiology, American University of Beirut Medical Center, Beirut, Lebanon; 2 Department of Internal Medicine, American University of Beirut Medical Center, Beirut, Lebanon; 3 Department of Neurology, American University of Beirut Medical Center, Beirut, Lebanon; 4 Department of Internal Medicine, Division of Hematology-Oncology, American University of Beirut Medical Center, Beirut, Lebanon

**Keywords:** case report, immune-checkpoint inhibitors, immunotherapy, myocarditis, rechallenge, renal cell carcinoma

## Abstract

Immune checkpoint inhibitors (ICIs) have revolutionized the practice of oncology, becoming a cornerstone treatment for many cancers. Nivolumab, an antibody-targeting programmed cell death protein-1 (PD-1), and ipilimumab, an antibody-targeting cytotoxic T-lymphocyte-associated protein 4 (CTLA-4), are two ICIs that, when combined, lead to improved treatment responses and enhanced survival rates. However, dual immunotherapy can come at the expense of increased incidence of autoimmune-related adverse events. The mortality rate of ICI-induced myocarditis can be high, and therapy rechallenge can pose a significant risk of recurrence and severe complications. There is no consensus regarding therapy rechallenge after myocarditis, and this decision should be made in a multidisciplinary discussion following a patient-centered approach. In our paper, we report the case of an adult patient with metastatic renal cell carcinoma who developed multiorgan toxicity, including severe myocarditis that required pacemaker implantation, after a single cycle of ipilimumab and nivolumab. Importantly, we also report the consequences on her cardiac and safety profile following ICI rechallenge.

## Introduction

Immune checkpoint inhibitors (ICIs) have revolutionized the practice of oncology and have become the mainstay treatment for a variety of malignancies. Nivolumab, an anti-programmed cell death protein-1 (PD-1) antibody, and ipilimumab, an anti-cytotoxic T-lymphocyte-associated protein-4 (CTLA-4) antibody, are two ICIs that, when used in conjunction, result in greater treatment response and increased survival. However, dual immunotherapy can come at the expense of increased incidence of autoimmune-related adverse events. The mortality rates of ICI-induced myocarditis can reach up to 46%, and therapy rechallenge can be associated with worsening cardiotoxicity and severe complications (1). There is no consensus regarding therapy rechallenge after myocarditis, and this decision should be made in a multidisciplinary discussion following a patient-centered approach.

We report the case of an adult patient with metastatic renal cell carcinoma who developed multiorgan toxicity, including severe myocarditis requiring pacemaker insertion, following a single course of ipilimumab and nivolumab. Importantly, we also report the consequences on her cardiac and safety profile following ICI rechallenge.

## Case presentation

**Figure 1:**
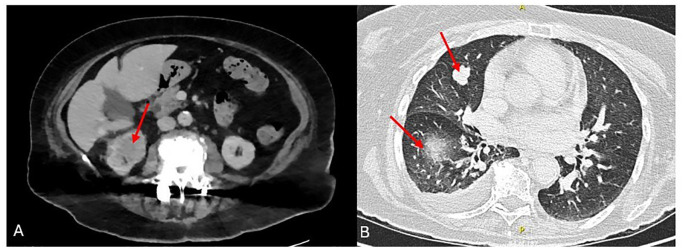
Computed tomography showing primary renal mass **(A)** and metastatic lung nodules **(B)** at diagnosis.

An 85-year-old female patient, without any known prior medical issues, was referred to us for evaluation of an incidental right kidney exophytic mass. Whole-body computed tomography (CT) scan showed a right kidney mass (7.8 × 6.5 × 5.5 cm) with heterogenous enhancement and necrotic core and multiple pulmonary nodules (**Figure 1**). The kidney biopsy showed clear cell renal cell carcinoma. Her Karnofsky performance score was 70%. She was considered as intermediate-risk metastatic disease for which a dual immunotherapy regimen with ipilimumab (1 mg/kg) and nivolumab (3 mg/kg) was commenced. The results of baseline lab tests including CBCD, complete metabolic profile, liver function tests, and TSH were within limits of normal. Baseline transthoracic echocardiography and ECG were also obtained and were within limits of normal.

**Figure 2:**
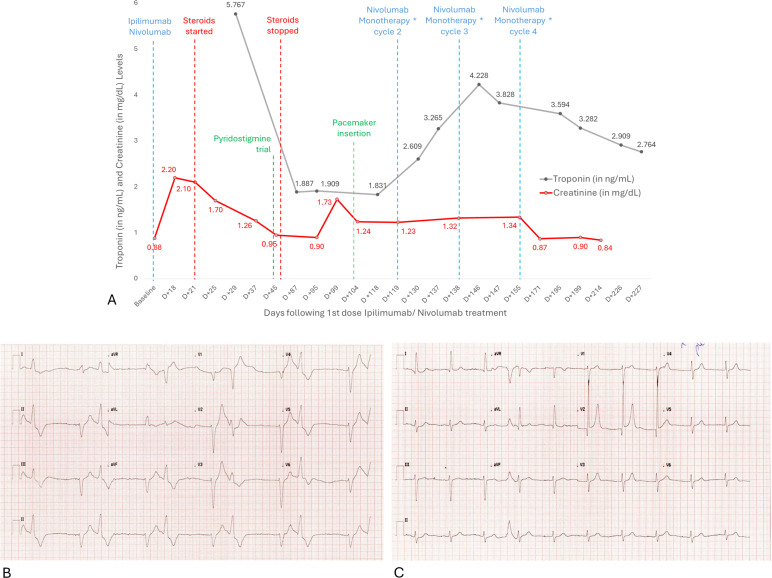
**(A)** The trend of troponin (grey) and creatinine (red) after the initial cycle of combined Ipilimumab/Nivolumab and following ICI rechallenge therapy with three cycles of Nivolumab monotherapy, showing stable creatinine levels throughout treatment but an increase in troponin following Nivolumab rechallenge. Interestingly, we note a progressive decrease in troponin during the surveillance period following cycle 1 and after discontinuation of treatment. **(B)** ECG on presentation showing high degree AV block. **(C)** ECG obtained 24 hours after the first dose of methylprednisolone 1 g given as an IV bolus, showing sinus rhythm with subtle p waves on leads II and III. *Nivolumab monotherapy restarted at 240 mg every 2 weeks. D, Days.

At 18 days following her first ipilimumab–nivolumab dose, the patient presented to the emergency department for lethargy and was found to have acute kidney injury (AKI) with a creatinine level of 2.20 mg/dL and grade 2 hepatitis. Fractional excretion of sodium (FeNa) was 3.3%, which was suggestive of intrinsic AKI. Our working diagnosis was kidney and liver injury secondary to immunotherapy, and she was given intravenous solumedrol (100 mg) once, followed by oral prednisone at 70 mg (1 mg/kg) daily, after which her creatinine level was progressively trending down (**Figure 2**).

At 4 days following discharge, she presented again for syncope and respiratory distress. The results of a workup revealed elevated cardiac biomarkers (hs-troponin T at 5.767 ng/mL, CPK at 1,191 iu/L, and pro-BNP at 5,408 pg/mL), worsening kidney injury (creatinine 1.97 mg/dL) despite being on daily prednisone (1 mg/kg), and normal electrolytes. An electrocardiogram showed complete atrioventricular block (heart rate of 40 bpm), and echocardiography showed a normal left ventricular systolic ejection fraction with no valvular disease. CT of chest without contrast showed essentially unchanged pulmonary nodules. The workup was highly suggestive of immunotherapy-related myocarditis and renal injury, for which she was admitted to the Cardiac Care Unit. The patient was switched to pulse steroid; methylprednisone was at 1 g daily for 3 days and then tapered to methylprednisone at 80 mg once daily on day 4, followed by an oral taper. Moreover, she was placed on bilevel positive airway pressure for her increased work of breathing. Given the patient’s advanced directives for no intubation and no invasive procedures, we continued with positive pressure ventilation and steroid therapy without intubation and without initiating plasmapheresis. A cardiac MRI could not be performed at that time given the patient’s critical respiratory status, advanced directives of no intubation and no invasive procedures, and non-availability of a MRI-compatible BIPAP machine. Within 24 h of pulse steroid administration, the patient went back into sinus rhythm (**Figure 2**).

Given her persistent respiratory distress, a neurology team was consulted. The patient had no diplopia, focal weaknesses, or sensory deficits but had bilateral ptosis and new-onset dysphagia to both solids and liquids. A full myasthenia gravis (MG) workup was ordered. Fatigability testing was difficult given the patient’s frail condition and the baseline of critical illness myopathy. Electromyography was pertinent for phrenic and myopathic changes. Her results revealed the presence of antibodies against acetylcholine receptors (anti-AchR). A trial of pyridostigmine (30 mg, three times daily) was attempted (on day +42 following the initial cycle of combined ipilimumab/nivolumab), with clinical improvement of her symptoms. At 18 days later, the patient was discharged home with complete resolution of her symptoms.

The patient was kept off immunotherapy on surveillance after a discussion of goals of care with her. At 2months later, the patient presented again to the emergency department for fatigue and foul-smelling urine. The workup revealed a new urinary tract infection, AKI (Cr = 1.7), and her ECG showed a recurrence of complete heart block with an accelerated junctional rhythm (HR 55–60 bpm). FeNA was <1%, suggesting a pre-renal etiology for her new AKI, keeping in mind her CT abdomen pelvis with IV contrast few days prior to her visit, raising the possibility of contrast-induced nephropathy as an additional factor. The patient was admitted for IV antibiotics and implantation of a dual chamber pacemaker after resolution of her infectious process. Subsequently, the patient started to have gradual improvement in her kidney function as evidenced by improved urine output and reduction in creatinine level (**Figure 2**). Following pacemaker insertion, the patient inquired regarding the safety and feasibility of resuming immunotherapy. A multidisciplinary meeting involving the oncology, cardiology, nephrology, neurology, and geriatrics teams was held to discuss immunotherapy rechallenge. The decision was to consider rechallenge with single-agent nivolumab at 2-week dosing and after a thorough explanation of the risks following the patient’s severe myocarditis and multi-organ toxicity. The patient consented on this approach, and nivolumab was restarted at 240 mg every 2 weeks with close monitoring of clinical status, including her renal, liver, and cardiac function. The patient tolerated the treatment well with no major changes in renal function or liver enzymes (**Figure 2**). However, her hs troponin T level was noted to increase again, reaching 3,265 ng/L. Her global longitudinal strain (GLS) decreased from -22.5%, at initial presentation, to -19.6% and stabilized during treatment. Her cardiac function (LVEF) was preserved and stable throughout the treatment course (**Table 1**). At 1 month following rechallenge, a follow-up CT of the abdomen with IV contrast showed reduction in the size of renal tumor to 5.1 × 4.7 cm (previously 6.5 × 6 cm). She received cycles 2–4 of the nivolumab monotherapy; all lab test results remained stable except for elevated hs troponin T. After discussing with the patient, a decision was made to withhold treatment, leading the hs troponin T levels to progressively trend down (**Figure 2**).

**Table 1:** Global Longitudinal Strain (GLS) and Left Ventricular Ejection Fraction (LVEF) patterns following Nivolumab monotherapy rechallenge.

	*GLS(%)*	*LVEF(%)*
At treatment initiation (day 0)	-22.5	55-60
During treatment (day + 139)	-20.0	55-60
During treatment (Day + 147)	-19.6	55-60

This table summarizes overall cardiac function for our patient following her ICI monotherapy rechallenge. Despite a stable LVEF, decreasing values for GLS were noted following monotherapy cycles.

## Discussion

While some immune-related adverse events (irAEs), such as myocarditis, are rare, it is even more uncommon for them to occur simultaneously with other organ involvement. The mechanism of irAEs is poorly understood but is thought to involve increased cytokine production and non-specific T-cell over-activation. Our patient developed multiorgan failure following her immunotherapy, including kidney and liver injury, new-onset MG, and, most importantly, severe myocarditis requiring pacemaker insertion.

Cardiovascular adverse events, although infrequent, can occur, and myocarditis remains the most common cardiotoxicity attributed to ICI use. The mechanisms of cardiotoxicity following ICI use are not fully understood, but infiltration of the myocardium with macrophages and CD4+ and CD8+ T-lymphocytes has been reported pathologically (2, 3). The overall incidence of ICI-related myocarditis is low, between 0.6% and to 2.1%, but the mortality rate is relatively high, between 25% and 50% in clinically symptomatic patients, which is significantly worse than other types of myocarditis (4). The overall high fatality rate can likely be explained by the lack of specific signs or symptoms, which makes the diagnosis challenging. Diagnosis can be achieved using cardiac biomarkers (troponin, pro BNP), echocardiography, and cardiac magnetic resonance imaging. In our case, the diagnosis of possible ICI-related myocarditis was based on a clinical continuum of multiorgan involvement and temporal correlation with ICI-related therapy. A definitive diagnosis could not be achieved due to logistical limitations pertaining to the cost and availability of MRI-compatible BIPAP and the patient’s advanced directives not to perform a cardiac biopsy or provide an advanced airway for respiratory support via mechanical ventilation. The initial prednisone dose of 1 mg/kg daily given to the patient resulted in only moderate improvement in her creatinine and could not prevent ICI-related myocarditis. The patient needed pulse steroids to revert her AV block and to regain her kidney and liver baseline function. This suggests that the guideline recommendations for ICI toxicity may need to be revisited when multiple organs are involved. While a prednisone dose of 1 mg/kg daily could be suitable for a patient with ICI-related AKI, a markedly higher dose may be needed when more than one organ is involved.

Immune-related myasthenia gravis (IrMG) is a rare but another serious complication that can occur in association with immune checkpoint inhibitors. It typically involves extraocular, bulbar, and limb muscles, leading to variable degrees of weakness that may progress to respiratory failure in 40%–65% of affected patients (5). In our patient, the diagnosis was supported by anti-AchR, but negative anti-MuSK and aldolase, and clinical findings suggestive of MG. It carries a worse prognosis and a higher mortality rate overall (29.8%) compared to classical MG (6%), likely related to the higher immunosuppression (6, 7). IrMG can occur as an exacerbation of a pre-existing MG or triggered by ICIs in patients with no prior history of MG (5). Treatment is similar to that of classical MG (6).

Treatment of ICI-related cardiac adverse events differs according to grades of toxicity. Current guidelines recommend discontinuing immunotherapy in cases of severe (grade 3) and life-threatening (grade 4) adverse events (8). Consequently, limited data is available regarding the relapse of ICI-induced myocarditis following rechallenge with ICIs. Although our patient remained asymptomatic and showed no changes in cardiac function (LVEF) following nivolumab rechallenge, an elevation in hs troponin T levels was observed, accompanied by a decline in her GLS, leading to the decision to withhold treatment. This caution regarding rechallenge may be viewed as a prudent preventive measure, considering the findings from a study by Simonaggio et al. (9). In their work, Simonaggio et al. examined the safety of ICI rechallenge in 93 patients with grade ≥2 immunological non-cardiac toxicities, including hepatitis, cutaneous reactions, pneumonitis, colitis, and arthritis. Among these, 43 patients were rechallenged with an anti-PD-1 or anti-PDL1 drug. The results showed that 55% of these individuals experienced immunological adverse events upon rechallenge, with 42.5% of cases involving a recurrence of the same type of toxicity, although none were more severe than the initial episodes (9).

Furthermore, in 11 published reports, 20 patients underwent immune checkpoint inhibitor rechallenge after developing ICI-induced myocarditis (**Table 2**). The initial myocarditis severity ranged between grades 2 to 5, and the first-line ICIs were anti PD-1 agents either used alone or in combination regimens. Upon rechallenge, most patients received another anti PD-1 agent, and the recurrence of myocarditis occurred in only 25% of the patients (5/20 people), with most patients having grade 1–3 myocarditis. All reported deaths were attributed to disease progression rather than myocarditis recurrence (8, 10–19). Despite some progress, this topic still requires significant attention in the literature and warrants further investigations. Nonetheless, rechallenging patients with ICI therapy in the presence of a history of myocarditis should be approached with utmost caution. It is worth mentioning that for our patient, a gradual drop in troponin levels was noted (**Figure 2**) during the surveillance period following the single dose of ipilimumab/nivolumab and again after the final discontinuation of her treatment, demonstrating the reversibility of myocardial injury following treatment discontinuation.

**Table 2:** Case reports and series of rechallenge of ICI after myocarditis.

Source	Number of patients rechallenged after myocarditis	ICI agents received initially (number of patients)	Grade of myocarditis prior to rechallenge	Rechallenge agent	Relapse of myocarditis after rechallenge	Outcome after rechallenge
Bailly, Guillaume et al., 2025 (10)	1	Pembrolizumab	Grade 3	Nivolumab	Yes	Resolution of arrhythmic events and biomarker abnormalities with complete cancer response on FDG-PET
Eldani, Céline et al., 2024 (11)	1	Anti-PD-1 monotherapy	Grade 0	Another anti-PD-1	No	No relapse of myocarditis
Menachery, Sherin M et al., 2023 (12)	1	Ipilimumab + Nivolumab	Grade 4	Pembrolizumab then switched to Nivolumab for progression	No	Patient eventually developed disease progression and was transitioned to hospice care
Shalata, Walid et al., 2023 (8)	1	Nivolumab	Grade 3	Nivolumab	No	Complete response to treatment
Coustal, Cyrille et al., 2023 (13)	6	Anti-PD-1 monotherapy or combination of anti-PD-1 with anti-CTLA-4	Grade 3-5	Anti PD-1	Yes in 1/6 patients (Grade 3)	3 patients achieved better oncological response, while the 3 others died from disease progression
Eslinger, Cody et al., 2023 (14)	1	Pembrolizumab	Grade 4	Nivolumab	No	Complete response after treatment for 1 year
Rossi, Valentina A et al., 2023 (15)	1	Anti-PD-1+ anti-LAG‐3 + anti-IDO‐1	Grade 1-2	Ipilimumab + Nivolumab	Yes (Grade 1)	Patient eventually developed disease progression and died after a year
Chen, Yeshan et al., 2022 (16)	1	Sintilimab with Fruquintinib	Grade 2	Zimberelimab	No	Remained in continuous remission
Chen, Xue et al., 2022 (17)	5	Anti-PD-1 or anti-PD-L1	Grade 2-3	Anti-PD-1 or anti-PD-L1	Yes in 1/5 patients (Grade 2)	1 patient died from disease progression
Shen, Li et al., 2021 (18)	1	Pembrolizumab	Grade 2	Pembrolizumab at reduced dose	Yes (Grade 2)	Also developed liver and kidney dysfunction (milder compared to first treatment) that were treated along with partial disease regression
Lee, Dae Hyun et al., 2020 (19)	1	Pembrolizumab	Grade 4	Pembrolizumab	No	Patient developed progressive disease and was ultimately transitioned to hospice care

ICI, Immune-checkpoint inhibitor; PD-1, Programmed Cell Death Receptor‐1; PD-L1, Programmed Cell Death Ligand-1; CTLA-4, Cytotoxic T-Lymphocyte-Associated protein 4; LAG-3, Lymphocyte-Activation Gene 3; IDO‐1, Indoleamine 2,3‐dioxygenase‐1‐inhibitor.

The strength of this study lies in its novelty, as it is one of the few articles that explore the outcomes of immunotherapy rechallenge after ICI-induced multiorgan damage. One limitation for our study is that it includes only one patient, as it is a case report; hence, further studies are needed. Another limitation is that our diagnosis was only probable myocarditis given the inability to perform a cardiac MRI and endomyocardial biopsy, which were not performed due to logistical constraints and the patient’s wishes to avoid invasive procedures. MRI was further precluded by the patient’s refusal of intubation and reliance on non-compatible BIPAP support.

Finally, if the patient needs to be rechallenged with immunotherapy, published data suggests considering monotherapy with very close cardiovascular monitoring and 2 weeks of dosing, mainly with anti‐PD‐1 agents as they carry the lowest risk of cardiotoxicity as shown in a retrospective analysis of the registry data (8). More data is lacking and further studies with more robust evidence on ICI rechallenge are still needed.

## Conclusion

In conclusion, we present the case of a patient treated with a combination of ipilimumab and nivolumab who developed myasthenia-gravis-like symptoms and a probable myocarditis with increasing troponins and complete AV block, necessitating a dual-chamber pacemaker implantation. After the partial recovery of symptoms, ICI monotherapy was resumed, achieving renal tumor shrinkage, but hs troponin T elevation was persistent, which led to discontinuation after four cycles. Rechallenge with ICIs remains a controversial topic, with recurrence of immune-related adverse events reported in ~55%, yet this may be considered selectively when oncologic benefit outweighs risk and with adequate surveillance.

### Patient’s perspective

Despite experiencing multi-organ failure after her first cycle of immunotherapy, our patient was determined to resume treatment to avoid leaving her malignancy untreated. Following treatment resumption, she continued to experience myocardial injury, as indicated by the decreasing GLS on echocardiography. This prompted us to discontinue treatment due to the ongoing damage. Our patient was extremely concerned about the potential future consequences and chose to halt the treatment.

## Data Availability

The original contributions presented in the study are included in the article/supplementary material. Further inquiries can be directed to the corresponding author.
